# Attitudes and Expectations regarding Dementia Risk Estimation among the General Population in Germany, Spain, and Switzerland

**DOI:** 10.1002/alz.092179

**Published:** 2025-01-09

**Authors:** Annika Baumeister, Constanze Hübner, Michelle Gerards, Yahveth Cantero‐Fortiz, Federica Ribaldi, Jule Sauer, Julia Braun, Mercè Boada, Giovanni B Frisoni, Frank Jessen, Ayda Rostamzadeh, Carolin Schwegler, Björn Schmitz‐Luhn, Christiane Woopen

**Affiliations:** ^1^ Center for Life Ethics, University of Bonn, Bonn Germany; ^2^ Department of Psychiatry and Psychotherapy, Medical Faculty, University of Cologne, Cologne Germany; ^3^ Ace Alzheimer Center Barcelona – International University of Catalunya (UIC), Barcelona Spain; ^4^ Laboratory of Neuroimaging of Aging (LANVIE), University of Geneva, Geneva Switzerland; ^5^ Geneva Memory Center, Department of Rehabilitation and Geriatrics, Geneva University Hospitals, Geneva Switzerland; ^6^ Network Center for Biomedical Research in Neurodegenerative Diseases (CIBERNED), Madrid Spain; ^7^ German Center for Neurodegenerative Diseases (DZNE), Bonn Germany; ^8^ Excellence Cluster on Cellular Stress Responses in Aging‐Associated Diseases (CECAD), University of Cologne, Cologne Germany; ^9^ Department of Psychiatry, University of Cologne, Medical Faculty, Cologne Germany; ^10^ Faculty of Arts and Humanities, University of Cologne, Cologne Germany

## Abstract

**Background:**

In recent years, research on risk estimation and early detection of Alzheimer’s disease (AD) advanced swiftly. Studies are investigating the likelihood of developing Alzheimer’s dementia (ADD), even during asymptomatic, preclinical, and prodromal stages of AD. Particular hope is pinned on blood‐based biomarkers as these are less invasive than other methods such as lumbar puncture and Positron Emission Tomography (PET)‐scan. Due to the rapid progress of research in this area, it is anticipated that risk estimation measures will be made available to the general population in the future. As part of the PreTAD project (The Predictive Turn in Alzheimer’s Disease: Ethical, Clinical, Linguistic, and Legal Aspects), our objectives were to investigate attitudes and expectations regarding ADD risk estimation in the general population.

**Method:**

We conducted online surveys within the general population (representative quota sample) in Germany (N = 1,000), Spain (N = 1,000), and Switzerland (N = 750). Among others, we assessed aspects influencing decision‐making regarding ADD risk estimation, accepted invasiveness of risk estimation measures, preferred information sources, and expected impact of a hypothetical high risk for ADD.

**Result:**

Preliminary analyses showed that 68% of the German population, 77% of the Spanish population, and 71% of the Swiss population would want to know their risk for developing ADD (rather yes/yes) with a preference for lower invasiveness in all countries (i.e. blood sampling). Physicians were stated as primary information sources regarding risk estimation. Most important aspects influencing decision‐making were existing possibilities to cure or prevent ADD (means > 3,7; 1 = no influence; 5 = strong influence). Individual risk knowledge was rather perceived as a chance to prepare for the future (means > 3.5, 1 = do not agree; 5 = agree) but was also expected to influence feelings of self (means > 3,2) or to cause psychological burden (means > 3,1).

**Conclusion:**

Acceptance for ADD risk estimation, particularly with low invasive measures, seems rather high in Germany, Spain, and Switzerland indicating promising opportunities for the future integration of such strategies into public health initiatives. Physicians, as primary information sources, should carefully consider individual attitudes and expectations regarding ADD risk estimation as risk disclosure may have several psychological and long‐term implications.

